# Feasibility Assessment of Telehealth‐Based Cancer Pain Management Through Machine Learning: A Prospective Clinical Study

**DOI:** 10.1155/prm/9211861

**Published:** 2026-05-16

**Authors:** Sergio Coluccia, Anna Crispo, Alessandro Ottaiano, Mariachiara Santorsola, Massimo A. Innamorato, Valentina Cerrone, Rosario De Feo, Vincenzo Cascella, Dalila Esposito, Maria Pia Bruno, Francesco Sabbatino, Gianluigi Franci, Alessandro Vittori, Marco Cascella

**Affiliations:** ^1^ Istituto Nazionale Tumori-IRCCS “Fondazione G. Pascale”, via M. Semmola 9, Naples, 80131, Italy; ^2^ Pain Unit, Department of Neuroscience, Santa Maria Delle Croci Hospital, AUSL Romagna, Ravenna, 48121, Italy, ausl.ra.it; ^3^ Department of Medicine, Surgery and Dentistry “Scuola Medica Salernitana”, University of Salerno, Baronissi, 84081, Italy, unisa.it; ^4^ School of Medicine, University of Pavia, Pavia, 27100, Italy, unipv.eu; ^5^ Department of Anesthesia, Critical Care and Pain Medicine, ARCO, Ospedale Pediatrico Bambino Gesù IRCCS, Rome, 00165, Italy, ausl.ra.it

## Abstract

**Background:**

Although telehealth strategies can be effectively adopted to manage cancer pain, identifying the optimal care pathway for tailoring interventions and allocating resources remains difficult. Artificial intelligence and machine learning (ML) may help clinicians develop more accurate strategies for predicting whether patients need remote consultations or in‐person evaluations.

**Methods:**

Data from two cohorts of cancer pain patients were analyzed. Variables included sociodemographic and clinical data, including age, sex, ECOG performance status, metastases, bone metastases, pain type, breakthrough cancer pain (BTCP), and rapid onset opioids (ROOs) therapy. The main outcome was the number of televisits (one versus multiple). For preprocessing, datasets from the two cohorts were harmonized by aligning variable definitions, coding schemes, and data formats. Six models were tested: logistic regression, random forest (RF), gradient boosting machine (GBM), support vector machine (SVM), k‐nearest neighbors (KNNs), and multilayer perceptron (MLP). Training and tuning used a 7‐repeated 5‐fold cross‐validation approach. Performance was evaluated on a hold‐out test set using *F*1‐score, accuracy, and AUC‐ROC. A sensitivity analysis with two scenarios was performed to verify the effects of class weighting and excluding the cohort variable.

**Results:**

The final dataset included 270 patients. No statistically significant associations were identified between the available variables and the number of televisits. *F*1‐scores across models ranged from 0.33 (RF) to 0.65 (MLP), accuracy from 0.45 (RF) to 0.55 (SVM), and AUC‐ROC from 0.43 (RF) to 0.65 (LR). DeLong tests showed no significant differences between algorithms (*p* > 0.05). Although the MLP achieved the highest *F*1‐score, it exhibited instability, with 91% of null *F*1‐scores. Incorporating class weights slightly improved SVM (*F*1 = 0.58 and AUC = 0.62) and LR (*F*1 = 0.53 and AUC = 0.63) though not significantly.

**Conclusion:**

Although no model demonstrated strong predictive power, this ML‐based framework shows the potential of using structured telemedicine data to model clinical workload and optimize follow‐up strategies in cancer pain care.

**Trial Registration:** ClinicalTrials.gov identifier: NCT04726228 and NCT07038434

## 1. Introduction

Cancer pain is one of the most common and debilitating symptoms across all types of cancer, significantly reducing patients’ quality of life [1]. Globally, cancer‐related pain affects approximately 55% of the patients undergoing active anticancer treatment and up to 70% of those with advanced or metastatic disease, with moderate‐to‐severe intensity reported in approximately half of the cases [2]. In Italy, it is estimated that over 60% of the patients with advanced cancer experience persistent pain [3]. Moreover, managing cancer‐related pain can often be challenging [3]. For example, many patients with advanced disease can experience difficulties in attending frequent clinic visits due to functional limitations, severe pain, and other debilitating symptoms that impair daily activities [4]. In this challenging clinical context, telehealth interventions can help bridge this gap by improving access to care and maintaining ongoing pain management [5]. Notably, recent evidence indicates that telemedicine‐based programs can reduce pain intensity and pain interference with daily life compared with usual in‐person care [6]. These findings highlight the feasibility of remote care options and the effectiveness and acceptability of these strategies as key components of cancer pain management.

However, implementing and optimizing telemedicine‐based pain care pathways pose several challenges that must be addressed. Given the need for a personalized care process, cancer pain management often requires multidisciplinary input and flexible adjustment of therapy [7]. Therefore, designing a care pathway that integrates both in‐person clinic visits and remote assessments is often challenging. Key issues include identifying suitable assessment tools for remote pain evaluation, implementing reliable symptom monitoring devices, and determining when additional interventions or in‐person exams are necessary [8]. Calibrated and dynamic care frameworks are thus necessary for timely clinical assessments, additional diagnostic tests, or procedures. In the lack of comprehensive guidelines or large‐scale trials, developing such a structured telemedicine pathway depends on early experiences and expert consensus. Although previous experiences demonstrated that a hybrid care model, combining remote visits with on‐site hospital care, can represent an effective and safe strategy for managing cancer pain, precise refinement of this process, also integrating patient‐reported feedback and clinical outcomes, is essential [9–11].

On these premises, artificial intelligence (AI) and machine learning (ML) can be used to analyze multiple patient‐related variables, such as demographic, clinical, and therapeutic metadata, to predict which patients will require more intensive follow‐up and, ultimately, identify those likely to need additional remote consultations or earlier in‐person visits [12]. We hypothesized that routinely collected demographic and clinical variables available during telemedicine‐based cancer pain management could be implemented to model the need for additional teleconsultations within a hybrid (remote/in‐person) care pathway. The primary aim of this study was to evaluate and compare the performance of different ML algorithms in predicting whether patients would require a single televisit or multiple televisits, using real‐world data derived from a structured telehealth program.

## 2. Materials and Methods

### 2.1. Study Population, Design, and Ethics

This study is based on a prospective cohort of adult cancer patients managed through a telehealth‐based pain management program at two tertiary referral centers in Italy (Naples and Salerno). Clinical data were collected prospectively during routine care, according to standardized telemedicine protocols, and subsequently analyzed retrospectively for ML investigations. This design was selected to preserve the real‐world nature of the data while enabling a structured and reproducible analytical approach, consistent with feasibility and exploratory ML studies in healthcare settings.

The local Medical Ethics Committees approved this study (protocol code 41/20 Oss; date of approval: 26 November 2020; Ethical Committee Campania 2, No 2024/28590, 3 April 2025), and all patients provided written informed consent. The investigation was conducted in accordance with the Declaration of Helsinki.

### 2.2. Telemedicine Care Framework

According to the hybrid care model, all patients underwent an initial in‐person visit before entering the telemedicine pathway. This first consultation was required for informed consent acquisition, comprehensive clinical and pain assessment, baseline data collection, and patient or caregiver training in the use of telemedicine tools. At the end of the first visit, remote follow‐up consultations were generally scheduled based on clinical judgment, while the care model also allowed for on‐demand teleconsultations and rapid in‐person reassessment in case of worsening symptoms or inadequate pain control [9, 10].

### 2.3. Clinical Trial Registration

https://www.clinicaltrials.gov/study/NCT04726228?cond=NCT04726228%rank=1; https://clinicaltrials.gov/study/NCT07038434. This manuscript was previously posted as a preprint to preprints.org: doi: DOI: 10.20944/preprints202510.1661.v1.

### 2.4. Datasets

Two cohorts of cancer patients, Cohort 1 recorded during 2021 and Cohort 2 in 2023/2025, were considered in the analysis. A single operator (Marco Cascella) performed the televisits for both cohorts. The same technology and operational processes were adopted across cohorts, including a shared IT infrastructure for telemedicine delivery and data management (Platform “Sinfonia,” Campania Region, Italy). The platform supports visit scheduling, secure audiovisual communication, structured clinical data collection, document exchange, and storage in compliance with privacy and data protection regulations.

Metadata concerning any single televisit was recorded. The included variables were age, sex, tumor site, Eastern Cooperative Oncology Group (ECOG) performance status, presence of metastasis, bone metastasis, pain (breakthrough cancer pain [BTCP], no BTCP), type of pain (nociceptive, neuropathic/mixed), and rapid opioid onset (ROO) therapy (no, yes). The time‐varying variables, such as ECOG, BTCP, pain type, and ROO therapy, were considered at the first televisit. All variables included in the present analysis were defined a priori, at the time of cohort design and data collection, based on clinical relevance and routine availability within the telemedicine pathway. Variable selection was not influenced by the results of previous ML analyses or by post hoc, outcome‐driven criteria.

Continuous measurements were shown as mean (standard deviation [SD]) and median (interquartile range [IQR]), while categorical measures were synthesized with absolute frequencies (percentages). Repositories are available at [13, 14].

### 2.5. Dataset Harmonization and Preprocessing

This initial step involved adapting two datasets. For Cohort 2, time‐varying information about variables was collected and had to be aligned with the format of the Cohort 1 dataset. Specifically, after transposing data into a wide format, we considered information on age, sex, ECOG, bone metastasis, BTCP pain, pain type, and ROO therapy at the first televisit. Although data on tumor sites and the presence of metastasis were also available, they were excluded from the analysis due to the small number of observations and their strong correlation with other features.

The final data included 280 cancer patients from Cohort 1 (*n* = 226) and Cohort 2 (*n* = 54). Since 10 observations containing missing values were removed, a total of 270 complete observations were considered for the subsequent analysis.

The number of televisits was recorded in discrete values and categorized into one or more televisits. One‐hot encoding was applied to categorical variables, while age, the only continuous measure, was standardized based on the values in the training set.

Although formal power calculations are not routinely performed in exploratory ML studies, a sample size justification was provided to contextualize the robustness of the available data. Assuming an infinite population and an estimated prevalence of patients requiring multiple televisits of 19%, a sample of 280 individuals allows estimation of this proportion with a margin of error of 4.6% at a 95% confidence level.

### 2.6. ML Algorithms

Our analysis aimed to classify observations with one or more televisits. To illustrate the full methods pipeline and ensure reproducibility, the analytical framework was developed in accordance with established guidelines for the application of supervised ML models [15, 16]. Five ML algorithms and a logistic regressor (LR) model, used as a reference model, were implemented. Random forest (RF) and gradient boosting machine (GBM) are tree‐based ensemble methods composed of multiple decision trees (DTs) whose outputs are aggregated to generate the final prediction. RF relies on bootstrap aggregating (bagging) to construct decorrelated trees based on different feature subsets, resulting in a stable algorithm capable of handling high‐dimensional data. Key RF hyperparameters include the number of trees (ntree), the number of randomly selected features at each split (mtry), and the minimum node size. The boosting learner is an alternative approach applied to the sequential construction of DTs in the GBM ensemble method. After creating an initial DT, the prediction error is gradually reduced by subsequent trees. The growth of these trees is regulated by the number of trees, the terminal node size, and the learning rate (shrinkage parameter). The support vector machine (SVM) aims to find the best hyperplane that separates observations. This hyperplane maximizes the distance between the hyperplane and the nearest data points from both classes. The optimal hyperplane maximizes the margin between classes, while allowing controlled misclassification through regularization. When nonlinear relationships are present between the outcome and features, input features are implicitly mapped into a higher‐dimensional feature space through a kernel function. In this study, a radial basis function (RBF) kernel was implemented. Relevant hyperparameters include the kernel parameter gamma and the regularization parameter (cost), which controls the trade‐off between margin maximization and classification error (higher values enforce stricter separation). These parameters are usually tuned within known value ranges [17]. The k‐nearest neighbors (KNNs) algorithm is widely used for classification, where a new data point is classified based on the majority class among its *k* closest neighbors. The hyperparameter *k* must be specified before running the algorithm. The multilayer perceptron (MLP) is a feedforward artificial neural network composed of interconnected layers of neurons. Each layer computes weighted linear combinations of inputs followed by a nonlinear activation function. Model training is performed through backpropagation, which iteratively updates network weights to minimize prediction errors. In this investigation, the hyperbolic tangent was adopted due to its greater numerical stability in the presence of outliers [18]. The network architecture included one or two hidden layers (HLs), with the first layer comprising between one and five neurons and the second, when present, mirroring the first. The learning rate governed the magnitude of weight updates during training.

### 2.7. Data Splitting, Exploratory Analysis, and ML Models Training and Selection

Data was split into a 75/25% training–test set, balanced on the combination of the number of televisits and cohort. An exploratory univariate analysis was performed to detect statistical differences across cohorts and showed crude associations between the number of televisits (one, more televisits) with the other variables. Tests included Pearson’s chi‐squared test for independence and Fisher’s exact test. The Benjamini–Hochberg correction was applied to deal with multiple tests.

For ML modeling, training was performed using a repeated K‐fold cross‐validation strategy. Specifically, for each algorithm, we adopted *R* = 7 repetitions and *K* = 5 folds. For each hyperparameter configuration, the training dataset was partitioned into five folds, with four folds (*n* = 165) used for model training and the remaining fold (*n* = 39) used for validation.

To ensure reproducibility, a fixed random seed based on the values of *K* and *R* was applied to govern data splitting. Observations were randomly assigned to folds using a stratified approach, balancing both the outcome variable (single vs. multiple televisits) and cohort membership. Model selection was performed by identifying, for each algorithm, the hyperparameter configuration yielding the best cross‐validated performance.

Performances were calculated via the *F*1‐score metric. The selected models were rerun on the whole train set (*n* = 204) and internally tested and compared with each other on the *F*1‐score, accuracy, and AUC‐ROC measures calculated on the test set (*n* = 66). The DeLong test was adopted to detect pair‐wise differences in terms of prediction performances, according to the AUC score [19].

A sensitivity analysis was performed to evaluate the robustness of model performance under alternative assumptions. Specifically, all ML pipelines were rerun under two different scenarios. In the first scenario, class weights were applied to account for imbalances in both the outcome variable (number of televisits) and cohort membership. In the second scenario, the cohort variable was removed from the feature set, resulting in an unbalanced dataset while preserving the same training–validation splitting strategy used in the primary analysis. For the MLP, direct specification of class weights was not supported by the implementation used. Therefore, an oversampling procedure was applied to the training data to approximate the effect of class weighting.

All tests and estimates were presented at the 95% confidence level. The analyses were implemented using the R software for statistical analysis, Version 4.3.2, and the following packages were included: *dplyr* for data manipulation, *gtsummary* for data presentation, *caret* for the performance metrics, *computation, randomForest, gbm, e1071, class,* and *neuralnet* for algorithm implementation, *purrr* and *pROC* for AUC‐ROC analysis, and *ggplot* to draw the figures. The workflow is available at [20].

## 3. Results and Discussion

### 3.1. Results

Demographic and clinical data were collected into two separate datasets. The two cohorts include 226 individuals from Cohort 1 and 54 from Cohort 2. The characteristics of the sample are described in Table 1. Mean age was 64.5 years (±12.4), half were males, and the most prevalent tumor site was gastrointestinal (20.7%), followed by breast (12.9%). Most patients suffered from metastatic disease (90%), whereas half of them had bone metastasis. Almost 40% suffered from BTCP, while a similar prevalence was observed in neuropathic or mixed pain. Sixty percent of the sample underwent ROO therapy. The median number of televisits was 1 (IQR: 1, 3), with 143 patients having 1 televisit and 9% having more than four (see Table 2, in the middle). When analyzing the difference between cohorts (not shown in tables), Cohort 2 had a significantly higher ECOG than Cohort 1 (*p* = 0.02), reported a higher prevalence of bone metastases (70.4% vs. 45.1%, *p* < 0.01), and suffered from nociceptive pain more than cohort 1 (78.8 vs. 59.1, *p* = 0.01).

**TABLE 1:** Descriptive statistics and univariable analysis: overall sample distribution over all available features, by cohort, and by number of televisits.

Characteristic	Overall	Univariate analysis by cohort			Univariate analysis by number of televisits		
	*N* = 280^1^	Cohort 1, *N* = 226^1^	Cohort 2, *N* = 54^1^	*p*‐value^2^	One, *N* = 143^1^	More, *N* = 137^1^	*p* value^2^
Number of televisits				0.423			
One	226 (80.7%)	111 (49.1%)	32 (59.3%)				
More	54 (19.3%)	115 (50.9%)	22 (40.7%)				
Number of televisits (numbers)^3^							
Mean (SD)	2.2 (2.1)	2.2 (1.9)	2.4 (2.8)				
Median (IQR)	1 (1, 3)	2 (1, 3)	1 (1, 3)				
Cohort							0.180
Cohort 1	226 (80.7%)				111 (77.6%)	115 (83.9%)	
Cohort 2	54 (19.3%)				32 (22.4%)	22 (16.1%)	
Age				0.333			0.726
Mean (SD)	64.5 (12.4)	64.1 (12.3)	66 (12.6)		66.5 (12.5)	62.4 (12)	
Median (IQR)	65 (57, 74)	65 (56, 74)	68.5 (58.3, 75.8)		68 (58, 76.0)	62.5 (54, 72)	
(Missing)	5	5	0		2	3	
Sex				0.226			0.073
Female	140 (50%)	109 (48.2%)	31 (57.4%)		79 (55.2%)	61 (44.5%)	
Male	140 (50%)	117 (51.8%)	23 (42.6%)		64 (44.8%)	76 (55.5%)	
ECOG (numbers)^3^							
Mean (SD)	2.5 (0.7)	2.4 (0.6)	3 (1)		2.5 (0.8)	2.5 (0.7)	
Median (IQR)	2 (2, 3)	2 (2, 3)	3 (2, 4)		2 (2, 3)	2.0 (2, 3)	
(Missing)	3	1	2		1	2	
ECOG (class)				0.019			0.692
1‐2	147 (53.1%)	127 (56.4%)	20 (38.5%)		77 (54.2%)	70 (51.9%)	
3‐4	130 (46.9%)	98 (43.6%)	32 (61.5%)		65 (45.8%)	65 (48.1%)	
(Missing)	3	1	2		1	2	
Tumor site^3^							
Bladder	18 (6.4%)	16 (7.1%)	2 (3.7%)		12 (8.4%)	6 (4.4%)	
Breast	36 (12.9%)	30 (13.3%)	6 (11.1%)		13 (9.1%)	23 (16.8%)	
Endocrine	20 (7.1%)	11 (4.9%)	9 (16.7%)		6 (4.2%)	14 (10.2%)	
Gastrointestinal	58 (20.7%)	45 (19.9%)	13 (24.1%)		38 (26.6%)	20 (14.6%)	
Gynecological	11 (3.9%)	10 (4.4%)	1 (1.9%)		5 (3.5%)	6 (4.4%)	
Head/neck	14 (5%)	14 (6.2%)	0 (0.0%)		6 (4.2%)	8 (5.8%)	
Kidney	6 (2.1%)	6 (2.7%)	0 (0.0%)		1 (0.7%)	5 (3.6%)	
Lung	39 (13.9%)	31 (13.7%)	8 (14.8%)		22 (15.4%)	17 (12.4%)	
Melanoma/skin	5 (1.8%)	5 (2.2%)	0 (0.0%)		3 (2.1%)	2 (1.5%)	
Prostate	22 (7.9%)	16 (7.1%)	3 (5.6%)		11 (7.7%)	8 (5.8%)	
Soft tissue/bones	32 (11.4%)	22 (9.7%)	0 (0.0%)		9 (6.3%)	13 (9.5%)	
Others	19 (6.8%)	20 (8.8%)	12 (22.2%)		17 (11.9%)	15 (10.9%)	
Metastasis				0.329			0.112
No	31 (11.1%)	23 (10.2%)	8 (14.8%)		20 (14.0%)	11 (8.0%)	
Yes	249 (88.9%)	203 (89.8%)	46 (85.2%)		123 (86.0%)	126 (92.0%)	
Bone metastasis				0.002			0.901
No	142 (50.7%)	125 (55.3%)	17 (31.5%)		72 (50.3%)	70 (51.1%)	
Yes	138 (49.3%)	101 (44.7%)	37 (68.5%)		71 (49.7%)	67 (48.9%)	
BTCP				0.083			0.252
No	169 (60.4%)	142 (62.8%)	27 (50%)		91 (63.6%)	78 (56.9%)	
Yes	111 (39.6%)	84 (37.2%)	27 (50%)		52 (36.4%)	59 (43.1%)	
Type of pain				0.011			0.086
Nociceptive	175 (62.7%)	133 (59.1%)	42 (77.8%)		96 (67.6%)	79 (57.7%)	
Neuropathic/mixed	104 (37.3%)	92 (40.9%)	12 (22.2%)		46 (32.4%)	58 (42.3%)	
(Missing)	1	1	0		1	0	
ROO therapy				0.826			0.691
No	110 (39.4%)	88 (39.1%)	22 (40.7%)		58 (40.6%)	52 (38.2%)	
Yes	169 (60.6%)	137 (60.9%)	32 (59.3%)		85 (59.4%)	84 (61.8%)	
(Missing)	1	1	0		0	1	

Abbreviations: BTCP, breakthrough cancer pain; ECOG, Eastern Cooperative Oncology Group; ROO, rapid onset opioid.

^1^ *n* (%).

^2^Pearson’s chi‐squared test, Wilcoxon rank sum test. The Benjamini–Hochberg correction for multiple testing.

^3^Not included in the multiple testing correction.

**TABLE 2:** Hyperparameters to optimize the model on the 7‐repeated 5‐cross validation technique.

	Hyperparameters/characteristics	Execution time to train and select (mins)	R package used for implementation
RF	• ntree: 20–100, step of 10 • mtry: 3–6 • nodesize: 9, 11, 17	0.86	*randomForest* (v. 4.7.1.1)

GBM	• ntree: 20–100, step of 10 • node size: 9, 11, 17 • shrink: 0.001, 0.005, and from 0.01 to 0.1, step of 0.01	3.39	*gbm* (version 2.2.2)

SVM	• gamma: 2 raised to the power of: from −15 to 3, step of 2 • c: 2 raised to the power of: from −5 to 15, step of 2	3.60	*e1071* (version 1.7.14)

KNN	*k* from 2 to 5	0.01	*class* (version 7.3.22)

MLP	• learning rate: 0.001, 0.005, and from 0.01 to 0.19, step of 0.02 • HL1: from 1 to 5 • HL2: from 0 and < HL1	231.30	*neuralnet* (version 1.44.2)

LR	logit link		*stats* (from *R* version 4.3.2)

Abbreviations: GBM, gradient boosting machine; KNN, k‐nearest neighbors; LR, logistic regression; MLP, multilayer perceptron; RF, random forest; SVM, support vector machine.

Nevertheless, no differences in the number of televisits were observed by cohort (*p* > 0.90). When analyzing the primary outcome, no statistically significant associations were observed between the number of televisits and the available set of features (Table 1). Nevertheless, trends toward an association were noted for sex and pain type. Specifically, the need for multiple televisits appeared more frequent among males than females (55.6% vs. 44.8%, *p* = 0.07), and patients requiring multiple televisits more commonly presented with neuropathic or mixed pain compared with those who underwent a single televisit (*p* = 0.09).

### 3.2. ML Modeling

Table 2 summarizes the hyperparameter grids and computational time required to optimize each ML algorithm using the 7‐repeated 5‐fold cross‐validation procedure. For each model, a range of key hyperparameters was explored to identify the configuration providing the best validation performance.

The RF and GBM models were tuned by varying the number of trees (ntree = 20–100), node size (9–17), and the number of features randomly sampled at each split (mtry = 3–6 for RF) or the shrinkage rate (shrink = 0.001–0.1 for GBM). The SVM was optimized over exponential grids of gamma (2^−15^ to 2^3^) and cost (2^−5^ to 2^15^). For the KNN algorithm, *k* varied from 2 to 5. The MLP network was tuned through combinations of learning rates (0.001–0.19, step 0.02) and the number of neurons in the first (HL1 = 1–5) and second HLs (HL2 < HL1). The LR model used a logit link as a reference.

Considering computational cost, ensemble and kernel‐based methods (GBM = 3.39 min; SVM = 3.60 min; and RF = 0.86 min) showed moderate training times, while the neural network (MLP) required substantially longer optimization (≈231 min). Simpler algorithms, such as KNN and LR, trained almost instantaneously.

The optimal hyperparameters and linked *F*1‐scores on the test set with accuracy and AUC‐ROC scores are shown in Table 3. Each model was retrained using the best configuration obtained from the 7‐repeated 5‐fold cross‐validation and then tested on unseen data (*n* = 66).

**TABLE 3:** Confusion matrices and associated performance metrics on the test set (*n* = 66).

	Optimal hyperparameters	TN	FN	FP	TP	*F*1‐score	Accuracy	AUC‐ROC
RF	ntree: 70; mtry: 3; node size: 9	19	19	15	19	0.43	0.49 (95% CI: 0.36, 0.61)	0.43
GBM	ntree: 80; node size: 11; shrink: 0.02	17	19	17	13	0.42	0.46 (0.33, 0.67)	0.42
SVM	gamma: 2; c: 0.125	18	14	16	18	0.55	0.55 (0.42, 0.61)	0.55
KNN	k: 3	18	18	16	14	0.45	0.48 (0.36, 0.61)	0.45
MLP	learning rate: 0.05; HL1: 4; HL2: 3	0	0	34	32	0.65	0.48 (0.36, 0.61)	0.65
LR		20	18	14	14	0.47	0.52 (0.39, 0.64)	0.47

*Note:* AUC‐ROC, area under the receiver operating characteristic curve; MLP, multilayer perceptron.

Abbreviations: FN, false negative; FP, false positive; GBM, gradient boosting machine; KNN, k‐nearest neighbors; LR, logistic regressor; RF, random forest; SVM, support vector machine; TN, true negative; TP, true positive.

Among ensemble methods, the RF achieved an *F*1‐score of 0.43 and an accuracy of 0.49 (95% CI: 0.36–0.61), while the GBM performed similarly (*F*1 = 0.42 and AUC = 0.42). The SVM with *γ* = 2 and cost = 0.125 showed a moderate improvement (*F*1 = 0.55; accuracy = 0.55; and AUC = 0.55). The KNN algorithm with *k* = 3 produced comparable results (*F*1 = 0.45; accuracy = 0.48; and AUC = 0.45).

Notably, the MLP, optimized with a learning rate of 0.05 and two HLs (HL1 = 4 and HL2 = 3), achieved the highest discrimination capacity (*F*1 = 0.65 and AUC = 0.65) although with relatively lower accuracy (0.48) due to class imbalance. Finally, the LR model yielded intermediate performance (*F*1 = 0.47; accuracy = 0.52; and AUC = 0.47), confirming its role as a stable but less flexible baseline classifier.

Despite the differences in mean values, no statistically significant difference among models was detected (DeLong test, *p* > 0.05), indicating comparable predictive capacities under the same data conditions.

**FIGURE 1:**
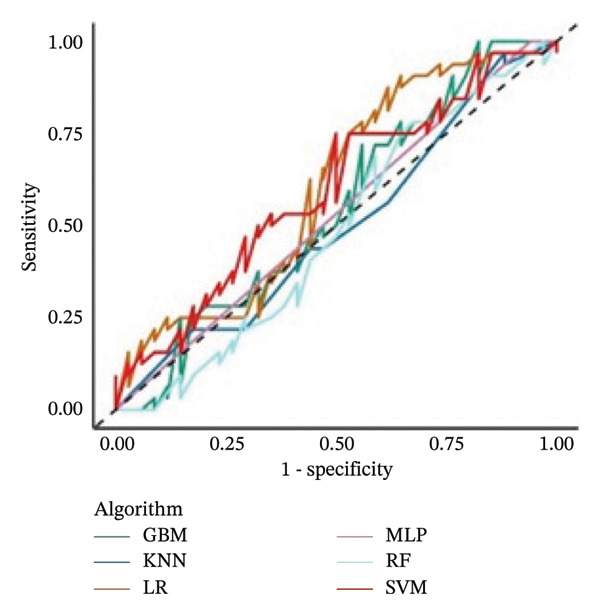
AUC‐ROC curves calculated on the test set (*n* = 66) of the six models. No significant differences were found across algorithms from the main analysis.

Comparisons across AUCs reported values from 0.43 (RF) to 0.65 (LR) (Figure 1). GBM was particularly unable to detect observations correctly, leading to an accuracy < 0.5, and to a statistically significantly worse AUC than SVM and LR (*p* value from the DeLong test = 0.04 and 0.03, respectively).

The sensitivity analysis did not reveal substantial differences in classification performance across models. However, additional computational time was required to train the MLP (approximately 6.8 h), due to the oversampling procedure applied to address class imbalance in the class‐weight subanalysis. In this context, the effective class weights were approximated through oversampling as follows: one televisit–Cohort 1 (*n* = 81; weight = 1.02), one televisit–Cohort 2 (*n* = 24; weight = 3.46), multiple televisits–Cohort 1 (*n* = 83; weight = 1.00), and multiple televisits–Cohort 2 (*n* = 16; weight = 5.19). In contrast, the computational time was reduced by about 1 h when the analysis was performed without considering the cohort among the features. Figure 2 reports the AUC‐ROCs from these additional analyses.

FIGURE 2AUC‐ROC curves calculated on the test set (*n* = 66) of the six models from the sensitivity analysis: (a) adding the weights procedure to the models and (b) removing the cohort among the features (see methods for the description). No significant differences were found across algorithms from the main analysis.

**Figure:**
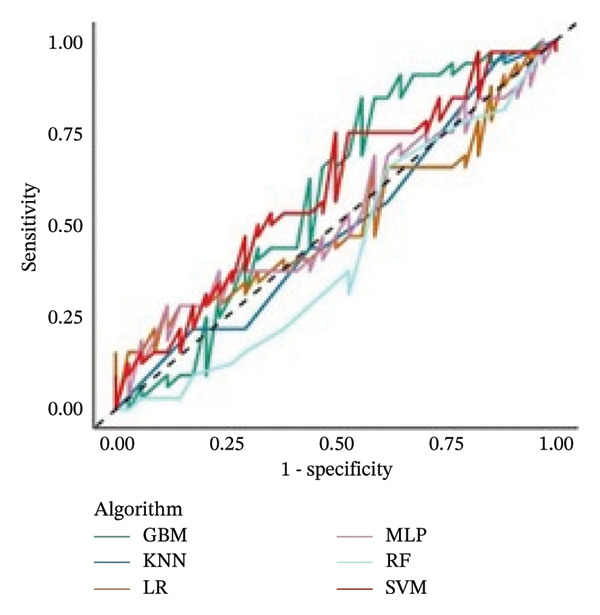
(a)

**Figure:**
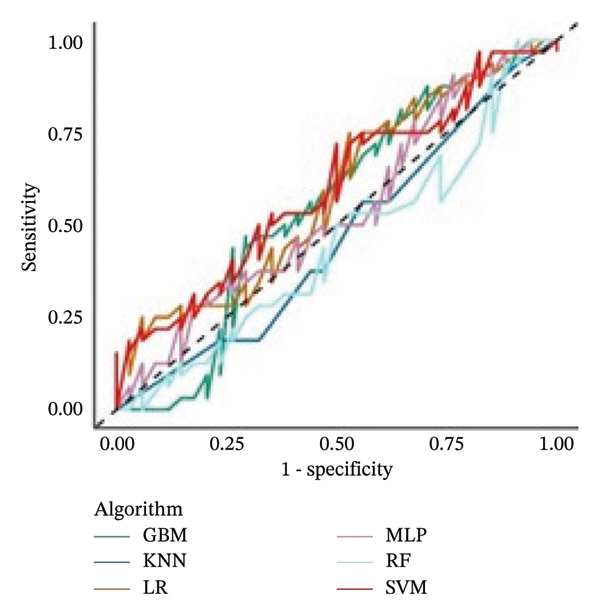
(b)

### 3.3. Discussion

Although telemedicine represents a suitable opportunity for care delivery, clinical practice indicates that scheduling and balancing in‐person and telehealth‐based consultations are often challenging [8, 11, 21]. In the complex context of cancer pain management, ML‐driven predictions can enable clinicians to tailor care interventions and allocate resources. In our previous ML investigation, we found that models identified specific factors that increased the likelihood of requiring multiple teleconsultations. These factors included younger patient age (< 55 years), certain cancer types (e.g., lung cancer), and the occurrence of BTCP episodes. The analysis also suggested that elderly patients (> 75 years) with advanced disease and bone metastases might benefit from closer telemedicine monitoring [22]. The present study shifts the focus from identifying individual predictive factors to comparing the performance and robustness of different ML algorithms within a unified analytical framework, thereby complementing our previous findings. The choice of study design and data sources reflects a deliberate methodological strategy. While ML models are highly sensitive to data quality and feature selection, the current literature does not offer strong evidence on which clinical variables are most informative for predicting telehealth utilization in cancer pain management. Moreover, our primary objective was to establish and validate a methodological framework for comparing different ML algorithms in a real‐world telemedicine setting. For obtaining methodological robustness and feasibility, we, therefore, relied on previously published clinical datasets from our group, ensuring a clear understanding of data provenance, quality, and underlying clinical processes and frameworks. Nevertheless, despite implementing rigorous validation procedures, model performance remained modest across all tested algorithms. These results may reflect intrinsic limitations of the dataset rather than methodological flaws.

A first critical issue relates to the risk of underfitting. In ML contexts, underfitting occurs when algorithms fail to learn sufficient patterns from the data, producing models that perform similarly to random classification [23]. This is a common problem in small and homogeneous datasets, especially when the outcome variable exhibits class imbalance or low variability [24]. Specifically, the dataset included a limited number of predictors, most of which were categorical and derived from baseline assessments. Consequently, the limited feature complexity may have affected the models’ ability to capture nonlinear relationships and hidden interactions among variables. However, the limited complexity of the dataset and the small number of clinically meaningful predictors likely constrained the ability of the algorithms to generalize. This is a key issue as cancer pain is influenced by multifactorial dynamics, including psychological, pharmacologic, and social variables, and probably they were not fully captured in this analysis [25]. Therefore, expanding future datasets to include pivotal information such as patient‐reported outcomes and analgesic adjustments, as well as clinical input obtained from wearable sensor data and digital technology tools, could substantially enhance predictive modeling [26]. Measures of pain intensity (e.g., NRS at rest or during movement), quality of life, treatment‐related adverse events, sleep disturbances, gastrointestinal symptoms, acute oncological complications, and psychological factors such as anxiety and depression are known to influence healthcare utilization and may play a critical role in determining the need for additional telemedicine or in‐person visits [27]. Their absence likely limited the ability of the models to capture clinically meaningful patterns and may have contributed to the modest predictive performance observed. Concerning opioid therapy, the use of ROOs was selected as a proxy variable to reflect treatment intensity and disease‐related pain severity in relation to the number of televisits. Nevertheless, we found that ROO use was not associated with the number of televisits (*p* = 0.8).

The presence of two distinct cohorts may have introduced heterogeneity that impaired model learning. Statistically significant differences were observed in ECOG performance status, pain type, and prevalence of bone metastases. These intercohort disparities may represent background differences not directly associated with the outcome (number of televisits), thereby acting as noise that limits pattern recognition. Similar findings have been described in ML studies using heterogeneous clinical populations, where variations in data source or clinical practice reduce algorithmic stability. They represent crucial gaps for AI deployment into clinical practice [28]. Andersen et al., for instance, discussed how variations in clinical data sources and operational settings can compromise the stability of AI and ML models, thereby requiring continuous monitoring practices and model updating strategies [29]. However, the inclusion of a comprehensive sensitivity analysis confirmed the robustness of the models across different training conditions. Techniques for handling class imbalance, such as class‐weight adjustment or oversampling of minority classes, have been extensively documented in the literature to mitigate bias due to class or cohort imbalance [30–32]. In our analysis, given the application of class‐weight adjustments and testing the exclusion of the cohort variable, potential sources of bias related to class imbalance and cohort heterogeneity were effectively mitigated. Therefore, model performance remained stable and was not driven by data distribution artifacts.

From a methodological perspective, other strengths could be highlighted. For example, the application of a repeated k‐fold cross‐validation scheme (7 × 5) represents a robust internal validation strategy, consistent with international guidelines such as the data, optimization, model and evaluation (DOME) approach, and the multidisciplinary recommendations for developing ML models in biomedicine [15, 16]. Moreover, the integration of multiple algorithms (tree‐based, kernel‐based, and neural models) within the same pipeline provides a comparative view of how different learning paradigms behave in a low‐dimensional telemedicine dataset.

Concerning the translational perspective of the proposed framework, given the availability of larger and more granular datasets, ML‐based care processes can be directly applied to predict high‐frequency teleconsultations, optimize clinician workload, and tailor follow‐up [33]. According to key objectives in modern telehealth oncology practice, these tools could assist healthcare providers in proactively identifying patients who might benefit from closer remote monitoring or careful in‐person evaluations [6]. Therefore, on these premises, future studies should aim to integrate temporal and textual features (e.g., free‐text notes and symptom trajectory), to evaluate model calibration and explainability using techniques such as SHAP or LIME, and to perform external validation on independent cohorts. Addressing these aspects will increase generalizability and clinical trustworthiness, aligning with emerging AI quality standards and the European AI Act framework [34, 35].

## 4. Conclusions

Despite limitations, this ML analysis confirms the feasibility of applying predictive analytics to telemedicine data for cancer pain management. Results highlight the importance of dataset quality, feature richness, and cohort homogeneity in developing reliable predictive models. Future research should expand data sources and exploit more sophisticated architectures to strengthen the integration of AI into personalized telehealth care.

## Author Contributions

Conceptualization: Sergio Coluccia, Marco Cascella, and Anna Crispo; methodology: Sergio Coluccia, Mariachiara Santorsola, and Francesco Sabbatino; software: Massimo A. Innamorato, Dalila Esposito, and Valentina Cerrone; validation: Alessandro Ottaiano, Maria Pia Bruno, and Rosario De Feo; formal analysis: Mariachiara Santorsola, Sergio Coluccia, and Alessandro Ottaiano; investigation: Dalila Esposito, Vincenzo Cascella, Alessandro Vittori, and Anna Crispo; resources: Rosario De Feo, Maria Pia Bruno, and Francesco Sabbatino; data curation: Massimo A. Innamorato, Dalila Esposito, and Mariachiara Santorsola; writing–original draft preparation: Sergio Coluccia, Alessandro Ottaiano, and Anna Crispo; writing–review and editing: Marco Cascella, Maria Pia Bruno, Alessandro Vittori, and Francesco Sabbatino; visualization: Vincenzo Cascella, Rosario De Feo, and Massimo A. Innamorato; supervision: Marco Cascella, Francesco Sabbatino, and Alessandro Ottaiano; project administration: Marco Cascella, Anna Crispo, Alessandro Vittori, and Rosario De Feo; funding acquisition: Marco Cascella, Alessandro Ottaiano, and Francesco Sabbatino.

## Funding

This research received no external funding. Open access publishing was facilitated by Universita Degli Studi di Salerno, as part of the Wiley‐CRUI‐CARE agreement.

## Disclosure

All authors have read and agreed to the published version of the manuscript. A preprint version of this manuscript has previously been published online (Coluccia et al., 2025 [36]).

## Conflicts of Interest

The authors declare no conflicts of interest.

## Data Availability

The data that support the findings of this study are available from the corresponding author upon reasonable request.
